# Elevation of serum lactate dehydrogenase in patients with pectus excavatum

**DOI:** 10.1186/1749-8090-9-75

**Published:** 2014-04-29

**Authors:** Jae Jun Kim, Chi Kyeong Kim, Hyung Joo Park, Jae Kil Park, Seok Whan Moon, Young Kyu Moon, Hyun Jung Kim

**Affiliations:** 1 Department of Thoracic and Cardiovascular Surgery, Ujeongbu St. Mary’s Hospital, The Catholic University of Korea College of Medicine, Seoul, Republic of Korea; 2 Department of Thoracic and Cardiovascular Surgery, St. Paul’s Hospital, The Catholic University of Korea College of Medicine, 620-56 Jeonnong-dong, Dongdaemun-ku, Seoul 130-709, Republic of Korea; 3 Department of Thoracic and Cardiovascular Surgery, Seoul St. Mary’s Hospital, The Catholic University of Korea College of Medicine, Seoul, Republic of Korea; 4 Department of laboratory medicine, Ujeongbu St. Mary’s Hospital, The Catholic University of Korea College of Medicine, Seoul, Republic of Korea

**Keywords:** Pectus excavatum, Lactate dehydrogenase, Nuss procedure

## Abstract

**Introduction:**

Pectus excavatum is the most common congenital chest wall deformity and the depression of the anterior chest wall, which compresses the internal organs. The aim of the present study is to investigate the effects of pectus excavatum on blood laboratory findings.

**Material and Methods:**

From March 2011 to December 2011, 71 patients with pectus excavatum who visited Seoul Saint Mary Hospital for Nuss procedure were reviewed and analyzed. The blood samples were routinely taken at the day before surgery and pectus bar removal was usually performed in 2 to 3 years after Nuss procedure. To investigate the effects on blood laboratory findings, preoperative routine blood laboratory data and postoperative changes of abnormal laboratory data were analyzed.

**Results:**

Only lactate dehydrogenase (LDH), one of 26 separate routine laboratory tests, was abnormal and significantly elevated than normal value (age <10, p = 0.008; age ≥10, p < 0.001). However, there was no significant correlation between LDH levels and severities of pectus excavatum. The symmetric subgroup had significantly higher LDH level than the asymmetric subgroup (p <0.001) and there was a significant decrease of LDH level after correction of deformity (p = 0.017).

**Conclusion:**

In conclusion, only LDH, one of the routine laboratory tests, was significantly elevated than normal value, which was thought to be caused by etiologies of pectus excavatum and the compression of the internal organs. Further studies on LDH including isoenzyme studies in patients with pectus excavatum will be needed, and these studies will provide a deeper and wider comprehension of pectus excavatum.

## Background

Pectus excavatum is the most common congenital chest wall deformity and the depression of the anterior chest wall, which compresses the lung and the heart [1-3]. Many studies on etiology, morphology and cardiopulmonary compromises have been performed. However, there are few studies on the blood laboratory findings of patients with pectus excavatum because most patients with pectus excavatum are so asymptomatic and otherwise healthy that no specific laboratory study is considered to be necessary [1-3]. However, we assumed that the etiologies or the compressions of internal organs including the heart and the lung could affect blood laboratory findings. The aim of the present study is to investigate the effects of pectus excavatum on blood laboratory findings. For this purpose, we compiled and analyzed preoperative routine blood laboratory results of the patients with pectus excavatum who had visited Seoul Saint Mary Hospital for Nuss operation, and analyzed postoperative changes of abnormal laboratory data.

## Methods

From March 2011 to December 2011, 71 patients with pectus excavatum who visited Seoul Saint Mary Hospital for Nuss procedure were reviewed and analyzed. Inclusion criteria for the present study were (1) preoperative diagnosis as pectus excavatum, (2) no combined other congenital anomaly or disease, (3) no other drug intake, and (4) operable status in aspect of general performance and laboratory results (5) ages from 5 to 19 years old (6) no definite inflammation or infection at the time of the surgery for Nuss procedure and pectus bar removal. To rule out the unknown effects on blood laboratory findings, we excluded the following cases: (1) any drug or medication intake (2) radiologic abnormalities except pectus excavatum, such as a tumor, inflammation or pneumonia. (3) any clinical symptoms, such as pain, fever, etc. The surgery for pectus bar removal was usually performed in 2 to 3 years after Nuss procedure, according to the patients’ ages and conditions. To investigate the effects of pectus excavatum on blood laboratory findings, Preoperative routine blood laboratory data and postoperative changes of abnormal laboratory data were analyzed. The blood samples were routinely taken at the day before surgery (Nuss procedure and pectus bar removal). The laboratory data taken at the day before Nuss procedure were defined as the preoperative ones and the laboratory data at the day before pectus bar removal were defined as the postoperative ones. Each patient underwent single a laboratory study consisting of 26 separate routine tests. The laboratory data were divided into gender and age. The reference laboratory ranges used in the present study were based on the current standardized test reference range at Seoul Saint Mary Hospital. Wherever applicable, age and gender- specific ranges were defined. These reference ranges are defined through varied methods, including healthy person for regular checkups and outside data accumulation. No patient’s data were excluded on the abnormal blood test results. The 26 laboratory tests included routine CBC (complete blood count) and blood chemistry. All laboratory tests were measured by standard methods using an auto analyzer (Hitachi 7600–210; Hitachi, Tokyo, Japan) and available assay kits (Sekisui Medical Co., Ltd., Tokyo, Japan). To decide abnormality of the laboratory test results, all data were analyzed with the simple T test and compared with the lower or upper limit of reference range. The comparisons between subgroup were performed with the ANCOVA (analysis of covariance). The relationship between severities of pectus excavatum and abnormal laboratory values was analyzed with the Pearson correlation test. The severity of pectus excavatum was defined as the Haller index, the chest wall depression index, and the cardiac compression index. The postoperative changes of abnormal laboratory data were analyzed with the paired T test. All data were analyzed using the Statistical Package of Social Sciences (SPSS) computer software program, version 18.0 (Chicago, IL, USA). A p- value < 0.05 was considered to be statistically significant. The approval from the Seoul Saint Mary's Hospital was obtained for the present study (KC12RISI0826)

## Results

**Figure 1:**
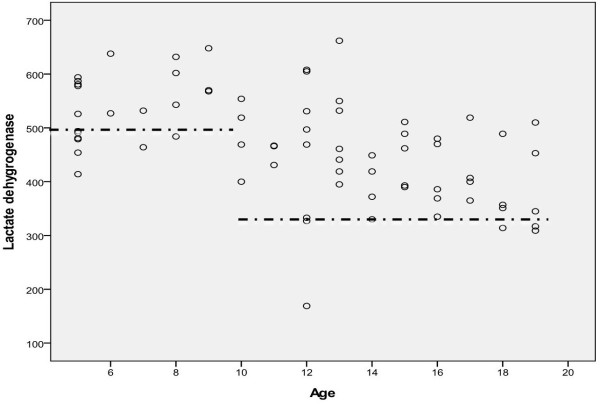
**Lactate dehydrogenase levels in patients with pectus excavatum.** Only lactate dehydrogenase was statistically abnormal and significantly elevated than normal value (age <10, p = 0.008; age ≥10, p < 0.001) and the other laboratory findings were statistically within normal range (Horizontal lines: upper normal limits of lactate dehydrogenase level according to ages).

We analyzed data from the overall 71 patients. The mean age was 11.92 years (range 5–19 year). Male and female patients were 55 and 16, respectively. Symmetric and asymmetric type was 30 and 41, respectively. Basic clinical characteristics of patients in the present study are summarized in Table 1. Out of the total 1,697 separate tests, the patients with pectus excavatum had 170 abnormal laboratory values. These laboratory results are summarized in Tables 2 and 3. Only lactate dehydrogenase (LDH), one of the 26 separate laboratory tests, was statistically abnormal and significantly elevated than normal value (age <10 p = 0.008; age ≥10 p < 0.001) (Table 4, Figure 1), and the other 25 separate laboratory findings were statistically within normal range. However, there was no significant correlation between LDH levels and severities of pectus excavatum (defined as Haller index, chest wall depression index, and cardiac compression index). A considerable number of patients’ hematocrit, creatine phosphokinase level also increased, but there was no statistical significance. Though there was no significant difference of severity between symmetric and asymmetric subgroup, the symmetric subgroup had significantly higher LDH level than the asymmetric subgroup (p <0.001). There was a significant decrease of LDH level after correction of compression or depression (p = 0.017) (Table 5).

**Table 1:** Basic clinical characteristics of the patients

	**Mean**	**SD**
Age (year)	11.92	4.48
Haller index	4.39	1.48
Chest wall compression index	2.61	1.23
Cardiac compression index	4.20	1.40

**Table 2:** Overall laboratory results (Complete blood count) of the patients

**Component measured**	**Age (year)**	**Sex**	**Reference range**	**Mean(SD)**	**Total studied number**	**Total abnormal value (%)**
White blood cell(/μL)	5-6		6000-15000	7826.15(1486.72)	13	7.7.
	7-12		4500-13500	7312.92(1463.93)	24	0
	13-19		5000-1000	5822.35(1024.23)	34	23.5
Hemoglobin (g/dL)	5-6		10.5-14.0	12.35(0.55)	13	0
	7-12		11.0-16.0	13.81(1.02)	24	4.2
	13-19	M	14.0-18.0	14.66(0.87)	31	12.9
		F	12.0-18.0	13.67(0.32)	3	0
Hematocrit (%)	5-6		33-42	35.86(1.09)	13	0
	7-12		34-40	40.14(3.07)	24	0
	13-19	M	42-52	42.40(2.34)	31	48.4
		F	37-47	39.40(1.13)	3	0
Platelet (×10^3^/ μL)			150-450	258.77(50.91)	71	0

**Table 3:** Overall laboratory results (Blood chemistry) of the patients

**Component measured**	**Age(year)**	**Sex**	**Reference range**	**Mean(SD)**	**Total studied number**	**Total abnormal value (%)**
Blood urea nitrogen(mg/dl)	5-14		5-18	12.20(2.96)	48	4.2
	15-19		7-18	13.06(3.02)	23	8.7
Creatinine (mg/dl)	5-14		0.3-1.0	0.54(0.13)	48	0
	15-19	M	0.6-1.2	0.84(0.15)	21	0
		F	0.5-1.1	0.60(0.06)	2	0
Total protein (g/dl)	5-7		6.1-7.9	6.91(0.50)	10	0
	8-12		6.4-8.1	7.11(0.38)	17	0
	13-19		6.6-8.2	6.95(0.51)	26	12.5
Albumin (g/dl)	5		3.9-5	4.46(0.16)	11	0
	6-		4.0-5.3	4.46(0.25)	60	1.7
Aspartate aminotransferase (U/L)	5-		14-40	24.18(5.04)	71	0
Alanine aminotransferase (U/L)			5-45	14.97(4.39)	71	0
Alkaline phosphatase (U/L)	5-9		134-386	263.29(172.56)	14	7.1
	10-15	M	116-483	266.14(106.71)	14	7.1
		F	93-386	224.11(84.21)	9	11.1
	16-	M	58-237	91.56(37.21)	16	12.5
		F	45-116	77.00	1	0
Total bilirubin (mg/dl)			0.47-1.58	0.59(0.40)	71	2.8
Direct bilirubin (mg/dl)			0.13-0.47	0.18(0.13)	71	4.2
Gamma glutamyl transpeptidase (U/L)	5-14		0-23	13.44(3.64)	32	3.1
	15-	M	11-50	21.17(6.13)	18	5.6
		F	7-32	12.50(0.71)	2	0
Uric acid (mg/dl)	5		1.7-5.8	3.73(1.00)	7	0
	6-11		2.2-6.6	4.05(0.86)	12	0
	12-	M	3.0-7.7	4.05(0.86)	27	3.7
		F	2.7-5.7	4.62(0.30)	6	0
Calcium (mg/dl)	5-14		8.8-10.8	9.51(0.33)	48	0
	15-19		8.4-10.2	9.35(0.30)	23	0
Phosphorous (mg/dl)	5-11		3.7-5.6	5.10(0.52)	20	15.0
	12-15		2.9-5.4	4.85(0.53)	16	12.5
	16-19		2.7-4.7	4.10(0.42)	17	5.9
Sodium (mEq/L)	-14		138-145	141.13(1.66)	48	4.2
	15-		136-146	140.65(1.90)	23	0
Potassium (mEq/L)			3.5-5.01	4.15(0.25)	71	0
Chloride (mEq/L)			98-106	103.83(1.94)	71	4.2
Lactate dehydrogenase (U/L)	5-9		150-500	540.32(64.78)	22	63.6
	10-19		120-330	434.61(91.90)	49	87.8
Creatine phosphokinase (U/L)			5-130	107.49(32.91)	71	21.1
Amylase (U/L)			48-176	97.59(32.95)	52	1.9
Magnesium (mg/dl)			1.9-2.5	2.11(0.14)	71	4.2
Total cholesterol (mg/dl)	5-19		-170	143.92(23.83)	51	15.7
Ammonia (mcg/dl)			20-80	77.48(19.82)	52	40.4

**Table 4:** Analysis of lactate dehydrogenase before Nuss procedure

	**Age(year)**	**Reference range**	**Mean(SD)**	**95% confidence interval of the difference**	**p-value**
Lactate dehydrogenase (U/L)	5-9	150-500	540.32(64.68)	11.64 ~ 68.99	0.008
	10-19	120-330	434.61(91.90)	78.22 ~ 131.01	< 0.001

**Table 5:** Analysis of postoperative lactate dehydrogenase changes of the patients

	**Mean(SD)**	**95% confidence interval**	**p- value**
Difference (U/L)	33.441(77.87)	6.27- 60.61	0.017
(preoperative- postoperative)			

## Discussion

Pectus excavatum is the most common congenital chest wall deformity and the depression of the anterior chest wall, which compresses the lung and heart [1-3]. Until now, nearly all previous studies have been performed to investigate its etiology, cardiopulmonary compromise, morphology and surgical techniques. However, because most patients with pectus excavatum are so asymptomatic and otherwise healthy that no specific laboratory study is considered to be necessary, there are few studies on the blood laboratory findings of patients with pectus excavatum [1-3]. We assumed that the etiologies of pectus excavatum or the compression of internal organs including the heart and the lung could affect the blood laboratory findings. To find out the effects of pectus excavatum on laboratory findings, we analyzed the routine laboratory tests, which were performed routinely before surgery (Nuss procedure and pectus bar removal). These routine laboratory tests were composed of 26 separate tests, including complete blood count and blood chemistry tests. Only lactate dehydrogenase (LDH), one of the 26 separate routine laboratory tests, had statistically abnormal value and the other 25 separate laboratory tests were statistically within normal range.

LDH is a cytoplasmic enzyme found in the cells of all major organs, including the heart, the liver, the kidneys, the skeletal muscle, the brain, red blood cells, and the lung [4,5]. It is responsible for converting lactic acid into pyruvic acid, an essential step in producing cellular energy [4]. Because LDH is present in almost all body tissues, LDH test is usually used to detect tissue damage or inflammation [6]. LDH level can be elevated when various conditions, for example, hemolysis, drug, infection, renal and liver disease, muscular dystrophy, various cancers or tumorous condition and inflammation [5,7-12]. Because the total serum LDH is a highly sensitive but nonspecific test [4-6], the isoenzyme of LDH test is used to localize the specific site of organs [4,6]. Also, determining LDH level combined with the results of other clinically important enzymes (aspartate aminotransferase, alanine aminotransferase, and creatine phosphokinase) provides more accurate decisions [10]. However, because the preoperative routine laboratory test did not include the LDH isoenzyme tests, the isoenzyme patterns in patients with pectus excavatum could not be analyzed. Also the present study showed there were no abnormal findings in aspartate aminotransferase, alanine aminotransferase, and creatine phosphokinase.

Reference ranges for total LDH vary from laboratory to laboratory [13]. In our hospital, the reference range for age under 10 is under 500 units/L, and the reference range for the age of 10 and older is under 330 units/L. Beside these conditions mentioned above, LDH level can be elevated in some clinical situations, such as heart failure and pneumonia [4,14]. However, to our best knowledge, there is no report or study on LDH elevation in patients with pectus excavatum yet. Of course, we considered the possibility that high LDH level might be due to difficulty experienced during collection or handling of the sample. Because the other laboratory results were all normal, and careful monitoring was taken, we could rule out the errors [15,16].

Evidently, the present study showed LDH level was significantly elevated than the upper normal limit without any correlation between LDH and severity of pectus excavatum (defined as Haller index, chest wall depression index, and cardiac compression index), and without other enzymes abnormalities (aspartate aminotransferase, alanine aminotransferase, and creatine phosphokinase). In addition, the symmetric subgroup had significantly higher LDH level than the asymmetric subgroup and LDH level was significantly decreased after the correction of deformity. We propose here two hypotheses from the above findings. Firstly, LDH elevation was caused by etiologies of pectus excavatum and the compression of the internal organs, which was demonstrated by irrelevance with severity of pectus excavatum and a significant decrease after correction of pectus excavatum. If LDH elevation was caused by the compression of internal organ alone, LDH level would be correlated with the degree of compression and normalized after correction of compression. Also if LDH elevation was not relevant to the compression of internal organ, there would be no decrease of LDH level after correction of pectus excavatum. Secondly, the musculoskeletal growth abnormality can elevate LDH level. If LDH elevation was caused by the musculoskeletal growth abnormality in patients with pectus excavatum, LDH elevation in patients with pectus excavatum can provide another evidence to support the hypothesis that the musculoskeletal growth abnormality is one of etiologies in patients with pectus excavatum. Therefore, we assumed that postoperative LDH were higher than normal value, since causes or etiologies of pectus excavatum could not be removed by Nuss procedure.

To find out the definite causes of LDH elevation, it is important to know from what tissue the LDH was released into the bloodstream, and isoenzyme pattern differences from normal. However, because the routine preoperative laboratory test did not include the isoenzyme of LDH test, the definite causes for LDH elevation are not discovered yet. Further studies on LDH, including isoenzyme pattern analysis, will be needed to investigate the definite causes of LDH elevation.

To our best knowledge, the present study is the first and the systemic study on routine laboratory evaluations. But there were a few limitations in this study. Firstly, the present study is a retrospective, a small size, and a single center study. The larger studies from multi-centers will be needed. Secondly, because the routine preoperative laboratory test did not include the isoenzyme of LDH test, we do not still know the definite causes for LDH elevation. Further studies on LDH, including isoenzyme pattern analysis, will be needed to find out the exact causes of LDH elevation.

## Conclusion

Only LDH, one of the routine laboratory tests, was significantly elevated than normal value, which was thought to be caused by etiologies of pectus excavatum and the compression of the internal organs. Further studies on LDH including an isoenzyme study in patients with pectus excavatum will be needed, and these studies will provide a deeper and wider comprehension of pectus excavatum.

## Abbreviations

LDH: Lactate dehydrogenase; CBC: Complete blood count; ANCOVA: Analysis of covariance.

## Competing interests

The authors declare that they have no competing interests.

## Authors’ contributions

JK carried out overall study design, data collection, the statistical analysis, and drafted the manuscript. CK carried out overall review of the paper and helped to draft the manuscript. HP participated in data collection. JP participated in data collection. SM, MY and HK participated in study design. All authors read and approved the final manuscript.
